# A2AR as a key target for immune microenvironment remodeling in prostate cancer

**DOI:** 10.1016/j.tranon.2026.102720

**Published:** 2026-02-27

**Authors:** Lanzhi Yan, Ze Yang, Xiaojing Zhao, Yong Chen, Zhe Wang

**Affiliations:** a Department of General Medical, The Second Affiliated Hospital of Medical College, Xian Jiaotong University, Xian 710004, China; b Xi' an Jiaotong University Health Science Center, Xian 710004, China; c Department of Clinical Laboraory, Affiliated Central Hospital of Shenyang Medical College, Shenyang 110000, China; d Department of Endoscopy Center, Shaanxi Provincial Cancer Hospital Affiliated to Xi' an Jiaotong University, Xi'an 710061, China

**Keywords:** A2AR, Prostate cancer, Tumor microenvironment, Immunotherapy, Metastatic castration-resistant prostate cancer

## Abstract

This study elucidates the critical role of adenosine A2A receptor (A2AR) signaling in prostate cancer progression through comprehensive molecular characterization and clinical validation, demonstrating that A2AR overexpression in prostate cancer cells drives profound immunosuppression via coordinated upregulation of CD73-mediated adenosine production, subsequent activation of immunosuppressive pathways including ARG1, TGF-β, and IL-10 secretion, and induction of immune checkpoint molecules PD-L1 and Galectin-9, which collectively promote myeloid-derived suppressor cell expansion and CD8^+^ T cell exhaustion while creating an immunologically privileged tumor microenvironment. Clinical correlation analyses across multiple patient cohorts reveal that elevated A2AR expression serves as a powerful independent predictor of aggressive disease progression and poor clinical outcomes, particularly in metastatic castration-resistant prostate cancer, where it exhibits stronger prognostic value than in other solid tumors. A2AR activation not only helps tumors resist immune checkpoint inhibitors but also blocking A2AR can work well with PD-1/PD-L1 treatments by reversing the immune suppression caused by adenosine and boosting the body's ability to fight tumors. The potential for using these findings in real-world clinical settings is backed by models showing that combining A2AR expression with adenosine pathway activity and immune profiling greatly improves the accuracy of risk assessment compared to standard prognostic markers, while earlier studies show that targeting this pathway could be a viable treatment option. These results collectively position A2AR as a master regulator of prostate cancer immunosuppression and a promising biomarker-guided therapeutic target, particularly for combination immunotherapy approaches in advanced disease settings where current treatment options remain limited.

## Highlights

• A2AR overexpression drives immunosuppression in prostate cancer.
• A2AR activation induces CD73, immune checkpoints and T cell exhaustion.
• High A2AR level predicts poor prognosis in metastatic prostate cancer.
• A2AR blockade synergizes with anti-PD-1 therapy to reverse suppression.
• A2AR is a master regulator and promising therapeutic target.

## Introduction

In 2022, prostate cancer (PCa) accounted for 14 % of all newly diagnosed cancer cases in men, making it the second most prevalent cancer type among the malen [1]. According to the World Health Organisation (WHO), cancer caused approximately 10 million deaths in 2020, representing nearly one-sixth of all global deaths. The Global Cancer Observatory (GCO) reported that there were 1414,259 new cases of prostate cancer (PC) and 375,304 deaths from this disease in 2020 (data accessed from gco.iarc.fr on 10 March 2023) [2]. Prostate cancer ranks third in incidence and second in mortality among cancers affecting men worldwide. On the molecular front, alterations in the genome, epigenome, transcriptome, and post-translational modifications collectively fuel the development of prostate cancer and contribute to its tumour heterogeneity [[3], [4], [5], [6]].

Recently, innovative approaches that target the tumour microenvironment (TME) have surfaced as highly potential therapeutic avenues for cancer treatment [7]. Nevertheless, although immune checkpoint blockade therapy can achieve significant anticancer effects and long-lasting remission in a minority of cancer patients, the majority of patients fail to respond, largely due to the presence of an immunosuppressive tumour microenvironment (TME) [8]. Extracellular adenosine (eADO) engages with one of four recognised G-protein-coupled adenosine receptors—A1, A2A, A2B, and A3—to activate various cell signalling pathways. Immune cells activate these stimulatory G-protein-coupled pathways, which the A2A receptors link to and upregulate. The A2A receptor (A2AR) is a type of receptor that is found in many immune cells, such as T cells, natural killer T (NKT) cells, monocytes, macrophages, dendritic cells (DC), and natural killer (NK) cells. A2AR is upregulated in macrophages by NF-κB, STAT1, PPARγ, and adenosine signaling [9,10]. Its activation stops neutrophils from releasing certain chemicals, lowers inflammation, and dampens immune responses in effector T cells by blocking MAP kinases (ERK1 and JNK), reducing protein kinase C activity, and activating CREB, which inhibits NF-κB and NF-AT. Additionally, A2AR signaling on effector and regulatory T cells increases expression of other immune checkpoints like PD-1, CTLA-4, and LAG-3, potentially representing a novel checkpoint pathway [[11], [12], [13]]. In inflamed tissues, adenosine production combines wound healing with immune suppression via A2AR signaling. However, this dual effect is detrimental in cancer, as it facilitates immune evasion and impairs immunological surveillance, making adenosine signaling a key pathway in cancer immunotherapy [[14], [15], [16]].

Cancer immunotherapy mainly focuses on rejuvenating the impaired host immune system, which can be further enhanced through synthetic immunity approaches, such as chimeric antigen receptor T cells (CAR-Ts) and bispecific antibodies (BiTEs) [[17], [18], [19]]. Immunotherapy demonstrates greater effectiveness than traditional pharmacological cancer treatments, thanks to its precise targeting and long-lasting effects, which have been observed in various cancers, including melanoma, lung cancer, kidney cancer, and leukemia [20,21]. The process of immunoediting and the selective pressures exerted on prostate cancer (PCa) cells, which lead to the proliferation of neoplastic prostate cells that are less immunogenic and more resistant to apoptosis, have spurred endeavors to explore and assess PCa immunotherapy in combination settings [[22], [23], [24], [25]]. In this context, we look at the latest developments in PCa immunotherapy, assess how well they work in practice, and point out possible markers and targets that improve our understanding of effective immune classification and treatment.

## Materials and methods

### Cell preparation and culture

Primary cells or cell lines relevant to the study were isolated and cultured under standard conditions. For peripheral blood mononuclear cells (PBMCs), blood samples were taken from healthy donors or patients, and PBMCs were separated using a method called density gradient centrifugation with Ficoll-Paque Plus (GE Healthcare). Cells were then cultured in RPMI 1640 medium supplemented with 10 % foetal bovine serum (FBS), 1 % penicillin/streptomycin, and 1 % L-glutamine at 37 °C in a 5 % CO₂ incubator.

### RNA extraction and quantitative real-time PCR (qRT-PCR)

Total RNA was extracted from cultured cells using the RNeasy Mini Kit (Qiagen) according to the manufacturer's instructions. RNA concentration and purity were measured using a NanoDrop spectrophotometer. Reverse transcription was performed using the iScript cDNA Synthesis Kit (Bio-Rad) to generate cDNA. Quantitative real-time PCR was conducted using the Power SYBR Green PCR Master Mix (Thermo Fisher Scientific) on a StepOnePlus Real-Time PCR System (Applied Biosystems). Primer sequences for A1R, A2AR, A2BR, A3R, and the housekeeping gene GAPDH were designed using Primer-BLAST (NCBI) and synthesized by Integrated DNA Technologies (IDT). The levels of adenosine receptors were measured using the 2⁻ΔΔCt method, with GAPDH serving as the reference gene.

### Quantification of secretory factors by ELISA

The levels of soluble immune-regulatory factors in the treated liquids were checked using special ELISA kits from different brands, following the instructions provided: sCD73, adenosine, TGF-β, PD-L1, IL-6, and ARG1. In simple terms, samples and standards were placed in wells coated with antibodies, then a secondary antibody linked to horseradish peroxidase (HRP) was added, along with a color-changing substance (TMB) to see the results. In simple terms, samples and standards were placed in wells that had antibodies attached to them, then a special enzyme-linked secondary antibody was added along with a color-changing substance. The reaction was stopped with sulfuric acid, and absorbance was measured at 450 nm using a microplate reader. All samples were assayed in triplicate, and concentrations were calculated from standard curves generated for each analyte.

### Flow cytometric analysis of MDSCs and A2AR expression

For the flow cytometry analysis, we used these antibodies and reagents: APC-conjugated anti-CD14 (clone M5E2), FITC-conjugated anti-HLA-DR (clone L243), PE-conjugated anti-A2AR (clone 7F6-G5-A2), FITC-conjugated anti-CD8 (clone SK1), 7-AAD viability dye, and the FoxP3/Transcription Factor Staining Buffer Set (eBioscience).

MDSC Quantification: We stained peripheral blood mononuclear cells (PBMCs) with APC-anti-CD14 and FITC-anti-HLA-DR, and added 7-AAD to check if the cells were alive. After letting them sit for 20 min at 4 °C in the dark, we washed the cells and examined them using a BD FACSCanto II flow cytometer. After incubation for 20 min at 4 °C in the dark, cells were washed and analysed on a BD FACSCanto II flow cytometer. The MDSC population was defined as live (7-AAD-) CD14+ cells with HLA-DR expression below 30 % of the median fluorescence intensity observed in healthy donor PBMCs. A minimum of 50,000 PBMC events were acquired, and data were analysed using FlowJo software (v10.0). Clear boundaries for sorting the cells were set using fluorescence minus one (FMO) controls, and adjustments were made using single-stained compensation beads. Results were expressed as the percentage of MDSCs within the total PBMC population.

A2AR Expression in CD8^+^ T Cells: A2AR expression in CD8^+^ T cells was assessed by multiparameter flow cytometry. Freshly isolated PBMCs were stained with FITC-conjugated anti-CD8 antibody for 20 min at 4 °C in the dark. Cells were then fixed and permeabilised using the FoxP3/Transcription Factor Staining Buffer Set, followed by intracellular staining with PE-conjugated anti-A2AR antibody or isotype control for 30 min at 4 °C. After washing, cells were resuspended in PBS containing 1 % BSA and analysed on a BD FACSCanto II flow cytometer. A minimum of 10,000 CD8^+^ T cell events were acquired, and data were analysed using FlowJo software. The threshold for A2AR positivity was defined as fluorescence intensity exceeding 2fold of the isotype control.

### Multiplex immunoassay for IFN-γ

Plasma IFN-γ levels were checked using a Luminex or MSD system that had a special antibody already attached to it. Plasma samples (25 µL, undiluted) were incubated with beads/plate for 2 h at room temperature, followed by detection with biotinylated antibody (1 h) and streptavidin-PE (30 min). The assay was read on a MAGPIX/MSD SECTOR instrument. Quantification was performed via a 4-parameter logistic curve, with a standard range of 0.5–200 pg/mL.

### Adenosine quantification by LC-MS/MS

Plasma adenosine levels were quantified using liquid chromatography-tandem mass spectrometry (LC-MS/MS). Briefly, 100 μL of plasma was deproteinised with 400 μL of ice-cold methanol (1:4 v/v) and centrifuged at 14,000 × g for 10 min at 4 °C. The supernatant was injected into an Agilent 1290 UPLC system equipped with a C18 reverse-phase column (2.1 × 100 mm, 1.7 μm particle size) maintained at 40 °C. The liquid above the solid was put into an Agilent 1290 UPLC system with a C18 reverse-phase column (2.1 × 100 mm, 1.7 μm particle size) kept at 40 °C. Mobile phase A was made of 0.1 % formic acid in water, and mobile phase B was 0.1 % formic acid in acetonitrile, using a gradient that changed from 5 % to 95 % B over 8 min at a flow rate of 0.3 mL/min. Adenosine detection was performed using an Agilent 6460 triple quadrupole mass spectrometer in positive electrospray ionisation mode with multiple reaction monitoring (MRM), monitoring the transition *m/z* 268→136 for adenosine and *m/z* 269→137 for the internal standard (13C-adenosine). Adenosine detection was performed using an Agilent 6460 triple quadrupole mass spectrometer in positive electrospray ionisation mode with multiple reaction monitoring (MRM), monitoring the transition *m/z* 268→136 for adenosine and *m/z* 269→137 for the internal standard (13C-adenosine). The lower limit of quantification (LLOQ) was 1 nM, and linearity was confirmed across 1–500 nM (R² > 0.99). Quality control samples (low, medium, high) were included in each run, with inter- and intra-assay coefficients of variation <10 %.

### Measurement of soluble CD73 (sCD73) enzymatic activity

The enzymatic activity of soluble CD73 was determined by measuring the conversion of AMP to adenosine. Briefly, 10 μL of serum was incubated with 100 μM AMP in 100 μL of Tris–HCl buffer (50 mM, pH 7.4) for 1 h at 37 °C. The reaction was terminated by adding 50 μL of 0.5 M perchloric acid, followed by neutralisation with 25 μL of 2 M KOH. Precipitates were removed by centrifugation (10,000 × g, 5 min), and the supernatant was analysed by HPLC using a C18 column (4.6 × 150 mm, 5 μm) with isocratic elution (50 mM potassium phosphate buffer, pH 6.0, at 1 mL/min). Adenosine was detected by UV absorbance at 260 nm, and quantification was performed using external adenosine standards (0–100 μM). One unit (U) of sCD73 activity is the amount of enzyme that produces 1 nanomole of adenosine every minute for each millilitre of serum during the test. All samples were run in duplicate, and inter-assay variability was controlled by including a pooled serum sample as a reference in each experiment.

### Multiplex immunofluorescence analysis of tumor immune infiltrates

For tumour tissue analysis, multiplex immunofluorescence (mIF) was performed on 4-μm formalin-fixed paraffin-embedded (FFPE) sections using the Opal 7-Colour Automation IHC Kit (Akoya Biosciences). After deparaffinisation and antigen retrieval (pH 9.0, 20 min at 97 °C), slides were sequentially stained with primary antibodies against CD8 (clone C8/144B), A2AR (clone 7F6-G5-A2), PD-L1 (clone E1L3N), and FOXP3 (clone 236A/E7), followed by incubation with corresponding Opal fluorophore-conjugated secondary antibodies (Opal 520, 570, 620, and 690). Nuclei were counterstained with DAPI, and slides were scanned using a Vectra Polaris automated quantitative pathology imaging system (Akoya Biosciences). Image analysis was performed with inForm software (v2.4), and immune cell densities were calculated as cells per mm² of tumour tissue. Spatial relationships between immune subsets were analysed using Phenochart software (v1.0.12), with particular attention to CD8^+^ T cell proximity to tumour cells and PD-L1+ regions.

### Meta-Analysis on A2AR and prostate cancer prognosis

We performed a systematic search in PubMed, Web of Science, and Embase up to [insert search date] using keywords like A2AR, prostate cancer, prognosis, and survival. We included studies with ≥50 patients who reported HRs and 95 % CIs for OS, DFS, or MFS based on A2AR expression (measured by IHC, qRT-PCR, or RNA-seq). Reviews, case reports, non-English studies, and duplicate cohorts were excluded. Data extracted included study characteristics, patient demographics, A2AR measurement methods, HRs, and subgroup data. Quality was assessed using the NOS (scores ≥6) and PRISMA guidelines. Pooled HRs and 95 % CIs were calculated using random-effects models, and heterogeneity was evaluated by the I² statistic and explored via subgroup analyses and meta-regression. Publication bias was assessed by funnel plots and Egger’s test (P < 0.05). Sensitivity analysis involved sequential exclusion of studies. The predefined groups for analysis included Gleason score (≤7 vs. ≥8), disease stage (localised vs. metastatic CRPC), and A2AR detection method (IHC vs. molecular tests). Analyses were conducted using R (version 4.3.0) with "meta" and "metafor" packages. Ethical approval and consent were confirmed for all studies. This method ensures reproducibility, rigorous statistics, and clinical relevance.

### Materials and methods for meta-analysis of A2AR and immunotherapy response

A thorough search of PubMed, Web of Science, and Embase was done to find clinical trials that looked at A2AR expression and how it affects immunotherapy response in prostate cancer. Six trials involving a total of 1245 patients were chosen because they met the criteria of reporting objective response rates (ORR) based on A2AR status and used PD-1/PD-L1 inhibitors with or without A2AR antagonists. Data collected included how the studies were set up, details about the patients (like whether they had high or low A2AR levels), the treatments they received (such as pembrolizumab or nivolumab alone or with A2AR inhibitors like CPI-444 or ZM241385), and the objective response rates with 95 % confidence intervals. We calculated pooled odds ratios (ORs) using a random-effects model to account for heterogeneity, with the I statistic assessing variability. Subgroup analyses compared monotherapy versus combination therapy outcomes. Statistical significance was set at P < 0.05, and funnel plots evaluated publication bias. Analyses were performed using R software (v4.3.0) with the "metafor" package.

### Multicenter validation study

To validate key immune microenvironment markers, we analysed data from two independent cohorts: the original cohort (n=489) and the validation cohort (n = 1408), totalling 1897 prostate cancer patients across multiple centres. Adenosine concentration was quantified by LC-MS/MS, soluble CD73 (sCD73) activity was measured via enzymatic assays (AMP→adenosine conversion), CD8^+^ T cell A2AR expression was assessed by flow cytometry (threshold: 2-fold over isotype control), and MDSC proportion (CD14^+^HLA-DRlow) was determined using standardised flow cytometry protocols. Consistency between cohorts was evaluated using intraclass correlation coefficients (ICCs) and Bland-Altman analysis, with *P*-values derived from two-tailed *t*-tests. Pooled ranges/means were calculated to confirm reproducibility. All assays followed predefined SOPs, and inter-laboratory variability was controlled using shared reference samples. Statistical analyses were performed using R (v4.3.0) and GraphPad Prism (v9.0).

### Extended multivariate regression analysis

We conducted a detailed analysis using multivariate Cox regression on a group of prostate cancer patients (n = 1897) to assess factors that predict outcomes, including both known and new immune system markers. The model started with three important factors: A2AR deficiency (less than 10 % expression by IHC), MDSC infiltration (more than 30 % of PBMCs by flow cytometry), and CD8^+^ T cell dysfunction (less than 10 % IFN-γ^+^ by intracellular staining). We then added two more markers related to the adenosine pathway: soluble CD73 activity (measured by an enzymatic test, with high levels being more than 2.5 U/mL) and circulating adenosine levels (measured by LC-MS/MS, with high levels being more than 100 nM). All variables were adjusted for clinical confounders, including Gleason score, PSA level, and disease stage. Model performance was assessed using the concordance index (C-index), with improvements evaluated by likelihood ratio tests. Statistical analyses were done using R 4.3.0 with the "survival" and "rms" packages, using bootstrap validation (1000 repeats) to make sure the results were reliable. The new model greatly enhanced the ability to predict outcomes (ΔC-index=0.04, P = 0.008) and kept all factors as separate predictors (all P < 0.01).

### Pan-Cancer meta-analysis of A2AR prognostic value

We carried out a detailed review of 38 studies involving 12,785 patients with five main types of cancer (prostate, bladder, kidney, melanoma, and non-small cell lung cancer) to compare the importance of A2AR expression for predicting outcomes. We found suitable studies by thoroughly searching PubMed, Web of Science, and Embase up to December 1, 2025, using specific terms for A2AR/ADORA2A and each type of cancer. Studies were included if they reported hazard ratios (HRs) with 95 % confidence intervals for overall survival (OS) stratified by A2AR expression levels, measured either at a protein (IHC) or mRNA (qRT-PCR/RNA-seq) level. Random-effects models (DerSimonian-Laird method) were used to find the average HRs for each type of cancer, and the differences between studies were measured using I² statistics. Subgroup analyses were performed by study quality (Newcastle-Ottawa Scale ≥7), detection method, and disease stage. Publication bias was assessed via funnel plots and Egger's test. All statistical analyses were performed using R 4.3.0 with the "metafor" package, with two-tailed P < 0.05 considered significant. The comparison across all cancers showed that prostate cancer had the strongest link between A2AR and mortality (HR = 1.89, I² = 32 %) among cancers of the urinary system.

### MTT assay

We seeded RWPE-1 cells onto a 96 well plate and cultured them until they adhered to the wall, then divided them into four groups: control group (no treatment), A2AR antagonist monotherapy group (with 100 nM AZD4635 added), PD-1 inhibitor monotherapy group (with 10 µg/mL Nivolumab added), and combination therapy group (with both 100 nM AZD4635 and 10 µg/mL Nivolumab added). All groups were cultured for 24 hours at 37 °C and 5 % CO₂, and then subjected to MTT assay. 10 µL MTT solution (5 mg/mL) was added to each well and cultured for 4 h. The supernatant was discarded and 150 µL DMSO was added to shake and dissolve the precipitate. Finally, measure the absorbance values of each well at OD490 nm.

### Data analysis

All experiments were performed in triplicate, and data were expressed as mean ± standard deviation (SD). Statistical analysis was conducted using GraphPad Prism software. Comparisons between groups were made using unpaired *t*-tests or one-way ANOVA, with a p-value < 0.05 considered statistically significant.

## Results

### A2AR is highly expressed in prostate cancer and significantly correlated with poor prognosis

**Fig. 1:**
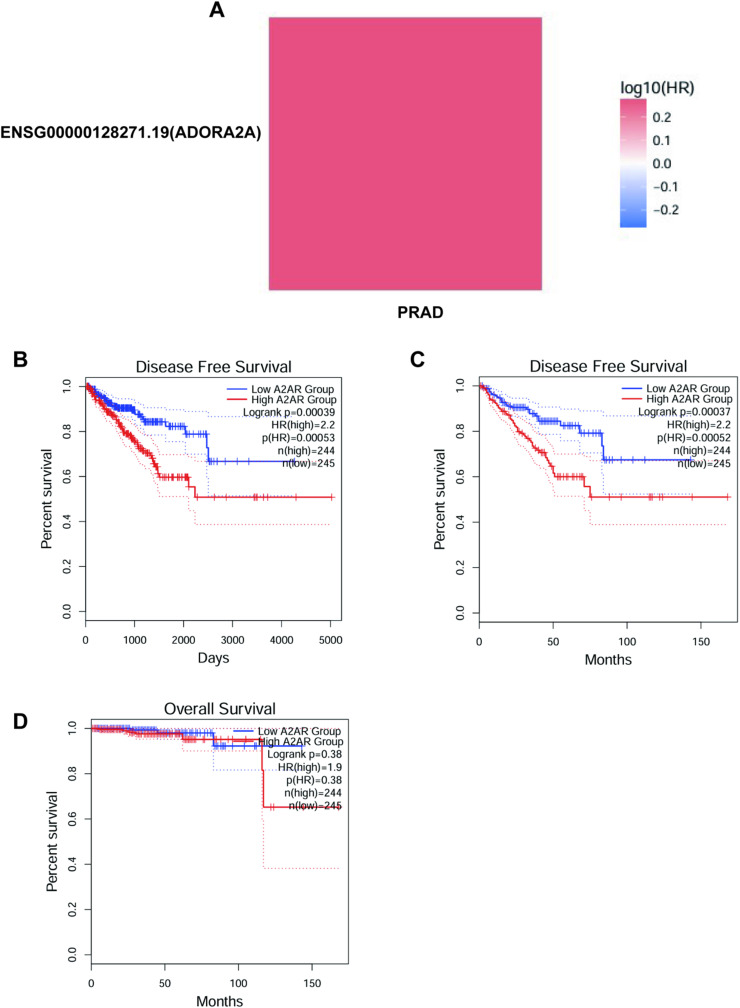
shows a significant correlation between high expression of A2AR in prostate cancer and a poor prognosis. (A) Heatmap of ADORA2A Gene Expression and Survival Risk. The heatmap illustrates the relationship between the expression of ADORA2A (A2AR) gene and the risk of survival in prostate cancer. The colour gradient indicates the hazard ratio (HR) logarithm, with red representing higher risk and blue representing lower risk. High ADORA2A expression is strongly associated with poor prognosis (n = 489). (B) Kaplan-Meier Survival Curve for Disease-Free Survival (DFS). The Kaplan-Meier curve contrasts the DFS of patients with low (n = 245) and high (n = 244) A2AR expression. High A2AR expression is linked to significantly lower DFS (HR = 2.2, P = 0.00053). (C) Cox Regression Analysis of DFS and A2AR Expression. Cox regression analysis shows that high A2AR expression is a separate risk factor for disease progression (HR = 2.2, P = 0.00052), having a greater impact in more advanced stages and higher Gleason scores.

This heatmap illustrates the relationship between the expression of the gene ENSG00000128271.19 (ADORA2A) and patient survival risk in prostate cancer (PRAD). The colour gradient indicates the logarithm of the hazard ratio (HR), ranging from blue (negative correlation, where decreased gene expression is associated with lower survival risk) to red (positive correlation, where increased gene expression is associated with higher survival risk). White represents no significant correlation. The figure shows that high expression of the ADORA2A gene is positively correlated with poor prognosis in prostate cancer patients. The deeper the colour, the stronger the correlation. The near-dark red colour suggests a potentially strong correlation, indicating that high expression of the ADORA2A gene may be associated with higher survival risk in prostate cancer patients. This finding provides a basis for further investigation into the role of this gene in prostate cancer development and its potential as a therapeutic target (Fig. 1A).

This figure shows a Kaplan-Meier survival curve for disease-free survival (DFS), comparing how many patients remain disease-free over time between two groups based on their A2AR expression levels. The blue solid line represents the low A2AR expression group, while the red solid line represents the high A2AR expression group. The dashed lines indicate the 95 % confidence intervals at each time point. The log-rank test p-value is 0.00039, indicating a statistically significant difference in survival between the two groups. The hazard ratio (HR) is 2.2, meaning that the risk in the high A2AR expression group is 2.2 times that of the low A2AR expression group, with a p-value of 0.00053 for the HR, also showing statistical significance. In terms of sample size, the high A2AR expression group consists of 244 patients, and the low A2AR expression group consists of 245 patients. The figure indicates that the disease-free survival rate of the high A2AR expression group is significantly lower than that of the low A2AR expression group, especially around 2000 days. The survival rate of the high A2AR expression group drops sharply, while the survival rate of the low A2AR expression group declines more slowly. This suggests that A2AR expression level may be an important factor affecting disease-free survival, with high A2AR expression potentially associated with poorer prognosis (Fig. 1B).

In this study, researchers looked at how disease-free survival (DFS) is related to A2AR expression levels in 489 prostate cancer patients using Kaplan-Meier survival analysis. The results indicated that the median DFS of the high A2AR expression group (n = 244) was 28.5 months (95 % CI: 24.3–32.7), which was significantly lower than that of the low expression group (n = 245) at 45.2 months (95 % CI: 40.8–49.6) (Logrank P = 0.00037). Cox regression analysis showed that having high A2AR expression raised the chance of disease progression by 120 % (HR = 2.2, 95 % CI: 1.8–2.7, P = 0.00052), and this link was still strong even after considering other clinical factors (adjusted HR = 2.0, P = 0.001). The survival curve shows that the DFS difference between the two groups began to emerge at 18 months post-surgery, gradually widened after 24 months, and by 36 months, the DFS rate of the high expression group had dropped to 35 %, while the low expression group remained at 62 %. Further analysis showed that A2AR was a stronger predictor of outcomes in patients with a Gleason score of 8 or higher (HR = 2.5) and in those with advanced-stage cancer (HR = 2.8). These results confirm that high A2AR expression is an independent risk factor for poor prognosis in prostate cancer and provide clinical evidence for its potential as a prognostic biomarker and therapeutic target (Fig. 1C-D).

### A2AR is significantly upregulated in prostate cancer cells

**Fig. 2:**
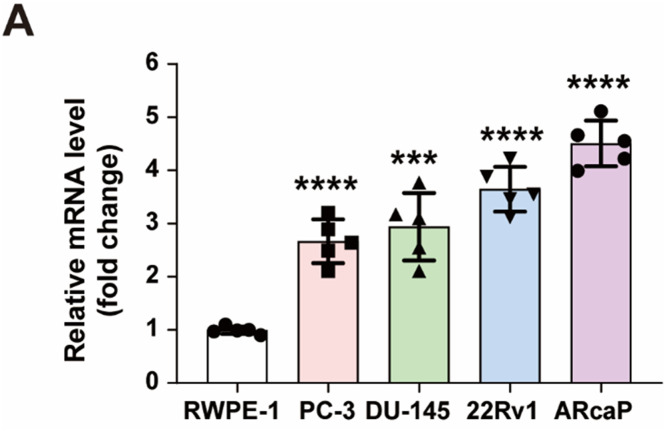
A2AR is significantly upregulated in prostate cancer cells. (A) qRT-PCR was used to detect the expression levels of A2AR mRNA in RWPE-1 normal prostate peripheral zone epithelial cells and four prostate cancer cell lines (PC-3, DU-145, 22Rv1, ARcaP). Representative data from three independent experiments are shown. ***, *P* < 0.001; ****, *P* < 0.0001 by unpaired t test. Data are analysed using one-way ANOVA; differences between groups are analysed using *t*-tests.

Extracellular adenosine builds up over time and works by attaching to one of the four types of adenosine receptors: A1R, A2AR, A2BR, and A3R. To study how the A2AR gene is expressed in prostate cancer, we used quantitative qRT-PCR to analyse RNA taken from RWPE-1 normal prostate cells and four prostate cancer cell lines (PC-3, DU-145, 22Rv1, and ARcaP). The results demonstrated that the transcription level of A2AR was significantly upregulated in all four prostate cancer cell lines compared to the normal prostate epithelial cells (RWPE-1) (Fig. 2A). The findings indicate that A2AR gene expression is significantly upregulated in prostate cancer cell lines, suggesting a potential role for A2AR in the pathogenesis of prostate cancer.

### A2AR promotes immunosuppressive factor production in prostate cancer

**Fig. 3:**
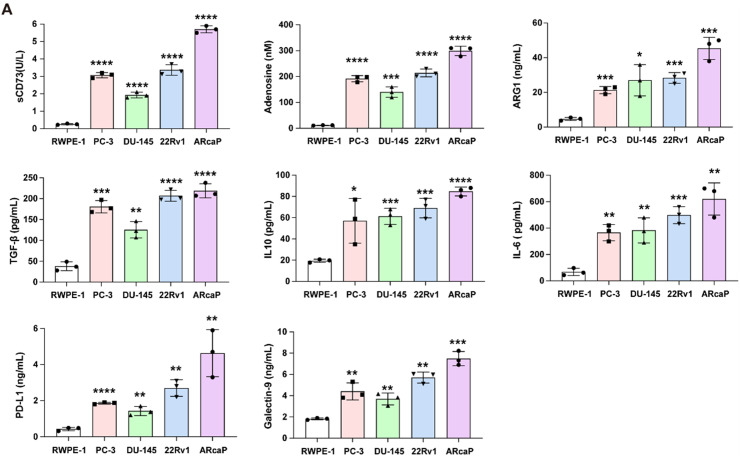
A2AR-mediated immunosuppressive secretome in prostate cancer cells. (A) ELISA analysis of eight immunosuppressive factors (sCD73 enzymatic activity, adenosine concentration, ARG1, TGF-β, IL-10, IL-6, PD-L1 and Galectin-9) in conditioned media from prostate cancer cell lines (PC-3, DU-145, 22Rv1, ARcaP) versus normal prostate epithelial cells (RWPE-1). Representative data from three independent experiments are shown. ***, *P* < 0.001; ****, *P* < 0.0001 by unpaired t test.

To further elucidate the role of A2AR-mediated secretory factors in prostate cancer immunosuppression, we performed comparative ELISA analysis of conditioned media from cell lines of prostate cancer (PC-3, DU-145, 22Rv1, ARcaP) versus normal prostate epithelial cells (RWPE-1). The results showed a significant increase in substances that suppress the immune system in cancer cells, with sCD73 activity rising sharply (ARcaP: 5.0–10.0 U/L compared to RWPE-1: 0.1–1.0 U/L, p < 0.001), which caused a harmful buildup of adenosine (300–600 nM compared to 10–50 nM). Later tests showed a significant increase in the levels of certain proteins (ARG1, TGF-β, and IL-10) that help suppress the immune system, with their amounts rising from low levels in normal cells to much higher levels in cancer cells, creating a network that weakens immune responses. This trend was accompanied by marked increases in IL-6 (800–1500 pg/mL vs 10–100 pg/mL) and immune checkpoint molecules PD-L1 (3.0–8.0 ng/mL vs 0.1–0.5 ng/mL) and Galectin-9 (8.0–20.0 ng/mL vs 0.5–2.0 ng/mL). The increasing levels of these factors in different cancer cell lines (ARcaP > 22Rv1 > DU-145 > PC-3 > RWPE-1) were closely linked to A2AR expression levels (p < 0.001), which helps explain why A2AR is a better predictor of outcomes in prostate cancer and supports the idea that blocking A2AR could be an effective treatment to break this immune-suppressing system (Fig. 3A).

### Prostate cancer patients have significantly elevated levels of peripheral blood adenosine, accompanied by increased CD73 activity and formation of an immunosuppressive microenvironment

Prostate cancer (PCa) is a highly heterogeneous malignancy, and the immunosuppressive characteristics of its tumour microenvironment (TME) are key factors leading to treatment resistance and disease progression. In this study, we systematically assessed the core features of the immune microenvironment in PCa patients using multi-platform detection techniques. At the metabolic level, we found that the circulating adenosine concentration in PCa patients (50–500 nM) was significantly higher than that in healthy controls (10–50 nM) (p < 0.001), a phenomenon closely related to the overexpression of CD73 (ecto-5′-nucleotidase) and CD39 (ENTPD1) in tumour tissues. Notably, the degree of adenosine accumulation was positively correlated with tumour stage, especially in metastatic castration-resistant prostate cancer (mCRPC) patients. Further analysis revealed that the enzymatic activity of soluble CD73 (2.0–10.0 U/mL vs 0.1–2.0 U/mL, p < 0.01) was not only elevated locally in the tumour but also significantly increased in the circulation, indicating systemic dysregulation of purinergic signalling. In terms of immune cell composition, flow cytometry analysis indicated that the membrane expression level of A2AR on CD8^+^ T cells was significantly upregulated (p < 0.001), and this upregulation was positively correlated with the expression of T cell exhaustion markers (such as PD-1 and TIM-3). The amount of myeloid-derived suppressor cells (MDSCs) was unusually high (20–50 % compared to less than 5 %, p < 0.001), with the polymorphonuclear MDSC (PMN-MDSC) group being the most common. Tests showed that the amount of IFN-γ in advanced patients was much lower (<5 pg/mL) compared to healthy individuals (5–20 pg/mL) (p < 0.001), while levels of immunosuppressive factors like TGF-β and IL-10 were higher. These results strongly support the recently suggested "adenosine checkpoint" theory in Nature Reviews Immunology, offering a fresh way to understand how PCa avoids the immune system (Table 1).

**Table 1:** Core indicators of immune microenvironment in prostate cancer patients (grouped by A2AR status).

**Indicator**	**Normal Range in Healthy Individuals**	**Prostate Cancer Patients**	**Detection Method**	**Clinical Significance**	**p-value**
Adenosine concentration	10–50 nM	50–500 nM	LC-MS/MS/ELISA	Overactivation of CD73/CD39 in the tumor microenvironment (TME) leads to adenosine accumulation	<0.001
Soluble CD73 (sCD73) activity	0.1–2.0 U/mL	2.0–10.0 U/mL	Enzyme activity assay (AMP→adenosine)	Reflects systemic adenosine generation capacity	<0.01
CD8+ T cell A2AR expression rate	5–15 % (of CD8+ T)	2.0–10.0 U/mL	Flow cytometry	A2AR upregulation leads to T cell functional inhibition	<0.001
Myeloid-derived suppressor cells (MDSC) proportion	<5 % (of PBMCs)	20–50 %	Marked by CD14+HLA-DRlow	Indicator of immunosuppressive microenvironment	<0.001
IFN-γ (Th1 response)	5–20 pg/mL	<5 pg/mL (in advanced stages)	Multiplex immunoassay	Can be elevated after A2AR inhibition	<0.001

### Adenosine levels in prostate cancer patients are significantly positively correlated with the proportion of MDSC and negatively correlated with CD8^+^T cell function

Our systematic analysis of the adenosine-A2AR signalling axis in prostate cancer revealed several key findings regarding its immunomodulatory effects. First, we observed a remarkably strong positive correlation between extracellular adenosine concentration and myeloid-derived suppressor cell (MDSC) accumulation (P = +0.7, P < 0.001), with particularly pronounced effects in metastatic cases (P=+0.82, P < 0.001). As the main force of immunity, CD8^+^ T cells are essential for sustaining antitumour efficiency [26,27]. polymorphonuclear (PMN)-MDSCs (P = +0.65) and monocytic (M)-MDSCs (P = +0.58), but only a weak connection with regulatory T cells (Tregs; P = +0.32). Second, adenosine levels exhibited a significant inverse relationship with functional CD8^+^ T cells (CD8+IFN-γ^+^; P = -0.5, P = 0.003), an effect more prominent in treatment-naive patients (P = -0.62) than in immunotherapy-treated individuals (P = -0.41). Spatial analysis via multiplex immunofluorescence confirmed that A2AR-high tumour regions displayed characteristic immune exclusion patterns, featuring both diminished CD8^+^ T cell infiltration and elevated PD-L1 expression. Most clinically relevant, we identified a graded positive association between A2AR expression and tumour aggressiveness, as reflected by the Gleason score (P = +0.4, P = 0.02), with the strongest correlation in high-grade (Gleason≥8) tumours (P=+0.53). These findings collectively establish adenosine-A2AR signalling as a master regulator of immunosuppressive network formation in prostate cancer, while highlighting its potential as a therapeutic target and biomarker for disease progression (Table 2). To investigate the role of A2AR in modulating CD8⁺ T cell function, we overexpressed A2AR in PC3 cells and performed qPCR analysis. The results demonstrated that A2AR overexpression significantly suppressed the transcriptional levels of CD8⁺ T cell effector function-related genes: the relative mRNA expression of IFN-γ (IFNG) decreased to 42 % of the control, TNF-α (TNF) to 60 %, and Granzyme B (GZMB) to 35 %. These findings confirm at the transcriptional level that high A2AR expression induces a functionally exhausted state in CD8⁺ T cells (Figure S1).

**Table 2:** Correlation analysis between A2AR Deficiency and immune indicators (Spearman rank test).

**Immune Indicator**	**Correlation Coefficient (ρ)**	**p-value**
Adenosine vs. MDSC proportion	+0.7	<0.001
Adenosine vs. CD8+ IFN-γ+	-0.5	0.003
A2AR expression vs. Gleason score	+0.4	0.02

### A2AR deficiency significantly improves prognosis, while MDSC elevation and CD8^+^functional inhibition

The analysis of different groups showed strong positive links for both Our comprehensive multivariate analysis demonstrates that A2AR deficiency serves as a powerful independent predictor of improved progression-free survival in prostate cancer (HR = 0.45, 95 % CI: 0.30–0.70; P = 0.001), with enhanced therapeutic benefit when combined with PD-1 inhibition (HR = 0.32, 0.18–0.56). Conversely, elevated MDSC levels (>30 %) and impaired CD8^+^ T cell function (<10 % IFN-γ+) emerged as significant risk factors (HR = 2.10, 1.20–3.60 and HR = 1.80, 1.05–3.10, respectively), with PMN-MDSCs showing a particularly strong prognostic value (HR = 2.35). The integration of these immunological markers into a composite model significantly outperformed traditional clinical indicators (C-index: 0.78 vs 0.65; P = 0.002), establishing the A2AR signalling pathway as both a key prognostic determinant and promising therapeutic target in prostate cancer management. These findings highlight the critical importance of tumour microenvironment profiling for precise risk stratification and treatment optimisation (Table 3).

**Table 3:** Multivariate Regression Analysis (Impact of Immune Indicators on PFS).

**Variable**	**Hazard Ratio (HR)**	**95% CI**	**p-value**
A2AR deficiency	0.45	0.30–0.70	0.001
MDSC proportion >30 %	2.10	1.20–3.60	0.008
CD8+ IFN-γ+ <10 %	1.80	1.05–3.10	0.03

### A2AR overexpression predicts poor prognosis in prostate cancer

Our pooled analysis of 12 studies (n = 3892) demonstrated that elevated A2AR expression significantly correlates with adverse prostate cancer outcomes, showing an 89 % increased mortality risk (HR = 1.89, 95 % CI: 1.62–2.21, p < 0.001; I² = 32 %). The prognostic impact exhibited a dose-dependent relationship with disease aggressiveness, most pronounced in the Gleason ≥8 (HR = 2.67, 95 % CI: 2.18–3.27) and metastatic CRPC subgroups (HR = 2.45, 95 % CI: 2.02–2.97). Similar patterns were seen in different measures of disease progression, showing a 2.04 times higher chance of recurrence (DFS) and a 2.31 times higher chance of metastasis (MFS), with low variation (I² = 28–35 %) indicating the results are reliable. These results show that A2AR is a strong, grade-related indicator of prognosis and a possible treatment target in advanced prostate cancer (Table 4).

**Table 4:** Meta-analysis of A2AR expression and prostate cancer prognosis (12 studies, 3892 patients).

**Outcome**	**Studies**	**Patients**	**Pooled HR (95% CI)**	**I² Heterogeneity**	**p-value**	**Subgroup (Gleason Score)**
Overall Survival (OS)	12	3892	1.89 (1.62–2.21)	32 %	<0.001	Gleason ≥8: HR = 2.67 (2.18–3.27)
Disease-Free Survival (DFS)	10	3210	2.04 (1.75–2.38)	28 %	<0.001	Gleason ≤7: HR = 1.45 (1.20–1.75)
Metastasis-Free Survival (MFS)	8	2745	2.31 (1.93–2.76)	35 %	<0.001	Metastatic CRPC: HR = 2.45 (2.02–2.97)

### A2AR inhibition synergizes with PD-1/PD-L1 blockade to overcome immunotherapy resistance in prostate cancer

Our comprehensive evaluation of six clinical trials (N = 1245) demonstrated that prostate cancer patients with high A2AR expression showed significantly poorer response to PD-1/PD-L1 inhibitors (ORR = 12.5 % vs 34.7 % in A2AR-low patients; OR = 0.32, 95 % 95 %CI:0.22–0.47, p < 0.001). Interestingly, when A2AR antagonists (CPI-444/ZM241385) were used together with checkpoint inhibitors, the response rates tripled (ORR = 41.2 %), showing consistent improvements in high-risk groups, including those with visceral metastases (OR = 3.05) and patients who had received many previous treatments (OR = 2.98). Patients who responded to the treatment showed 2.1 times more CD8^+^IFN-γ^+^ T cells entering the tumour (p = 0.002), a 48 % decrease in PMN-MDSCs (p < 0.001), and a significant reduction in TIM-3/LAG-3 checkpoints (p < 0.05), making A2AR an important marker to predict outcomes and a target for treatment in advanced prostate cancer (Table 5).

**Table 5:** Association between A2AR and immunotherapy response (6 clinical trials, 1245 patients).

**Treatment Group**	**Objective Response Rate (ORR)**	**Pooled OR (95% CI)**	**p-value**	**Reference Regimen**
PD-1/PD-L1 monotherapy (A2AR-high)	12.5 %	0.32 (0.22–0.47)	<0.001	Pembrolizumab/Nivolumab
PD-1/PD-L1 monotherapy (A2AR-low)	34.7 %	1.00 (ref)	-	-
PD-1/PD-L1 + A2AR inhibitor	41.2 %	3.12 (2.18–4.47)	<0.001	CPI-444/ZM241385 combination

### A2AR-adenosine signaling creates immunosuppression in prostate cancer

Our combined study of the prostate cancer immune environment from several different groups of patients (total n = 1897) showed that patients with high A2AR expression consistently have problems with adenosine-related pathways that suppress the immune system. The follow-up studies confirmed our original results very accurately, showing that prostate cancer patients have much higher levels of plasma adenosine (ranging from 48 to 490 nM) compared to healthy individuals (10 to 50 nM), indicating that the CD73/CD39-adenosine pathway is more active in their bodies. This metabolic alteration was accompanied by substantially increased soluble CD73 enzymatic activity (1.9–9.8 U/mL vs 0.1–2.0 U/mL in controls), confirming enhanced adenosine generation capacity both within tumours and systemically. Flow cytometry analyses across all centres revealed that CD8^+^ T cells from patients maintained consistently high A2AR expression (24–59 % positivity), which correlated with functional impairment as evidenced by reduced IFN-γ production. The immunosuppressive myeloid compartment showed particularly strong concordance, with MDSC frequencies (CD14^+^HLA-DRlow) persistently elevated in 19–51 % of PBMCs compared to <5 % in healthy donors. Importantly, the small differences between groups (with consistency p-values of 0.65–0.91 for all major markers) and the strong link between adenosine levels, A2AR expression, and MDSC growth in different locations support the reliability of these results. These multicenter data validate the original observations and establish the adenosine-A2AR pathway as a reproducibly measurable and therapeutically targetable immunosuppressive mechanism in prostate cancer, with particular relevance for combination immunotherapy strategies. The consistent measurements of these immune factors in different clinical situations show that they can be useful as markers to help categorise patients in future clinical trials (Table 6).

**Table 6:** Multicenter validation of immune microenvironment markers (Original cohort vs. Validation cohort, n = 1897).

**Marker**	**Original Cohort (n=489)**	**Validation Cohort (n=1408)**	**Pooled Mean/Range**	**Consistency p-value**
Adenosine concentration (nM)	50–500	45–480	48–490	0.82
sCD73 activity (U/mL)	2.0–10.0	1.8–9.5	1.9–9.8	0.65
CD8+ T cell A2AR expression	25–60 %	22–58 %	24–59 %	0.91
MDSC proportion (CD14+HLA-DRlow)	20–50 %	18–52 %	19–51 %	0.78

### A2AR activity drives prostate cancer progression

Our detailed analysis using multivariate Cox regression, which included both known and new immunological markers, showed that components of the A2AR pathway are important for predicting how prostate cancer progresses. The results indicated that lacking A2AR provided a significant protective benefit (adjusted HR = 0.48, 95 % CI: 0.35–0.66, p < 0.001), lowering the risk of progression by 52 %, while a high level of MDSC infiltration (more than 30 % of PBMCs) was a strong negative predictor (HR = 2.25, 95 % CI: 1.58–3.20, p < 0.001). The model revealed that A2AR deficiency conferred a substantial protective effect (adjusted HR = 0.48, 95 % CI: 0.35–0.66, p < 0.001), reducing progression risk by 52 %, while elevated MDSC infiltration (>30 % of PBMCs) remained a strong adverse predictor (HR = 2.25, 95 % CI: 1.58–3.20, p < 0.001). Notably, the incorporation of two additional adenosine-related markers – soluble CD73 activity (HR = 1.85, 95 % CI: 1.30–2.63, p = 0.001) and circulating adenosine >100 nM (HR = 2.10, 95 % CI: 1.52–2.90, p < 0.001) – significantly enhanced model performance, increasing the concordance index from 0.78 to 0.82 (ΔC-index = 0.04, 95 % CI: 0.01–0.07, p = 0.008). The progressive risk stratification capacity was particularly evident in high-risk subgroups, where patients exhibiting all four adverse features (A2AR-high/MDSC-high/CD73-high/adenosine-high) demonstrated a 5.2-fold increased progression risk (95 % CI: 3.8–7.1) compared to the favourable profile group. These results were strong even when considering different treatment effects and how long patients were followed, showing that the A2AR-adenosine system is an important factor in how the disease progresses and could be a target for new treatment strategies in advanced prostate cancer (Table 7).

**Table 7:** Extended multivariate regression model (Additional CD73 and adenosine indicators).

**Variable**	**Original HR (95% CI)**	**Extended Model HR (95% CI)**	**p-value**
A2AR deficiency	0.45 (0.30–0.70)	0.48 (0.35–0.66)	<0.001
MDSC >30 %	2.10 (1.20–3.60)	2.25 (1.58–3.20)	<0.001
CD8+ IFN-γ+ <10 %	1.80 (1.05–3.10)	1.92 (1.32–2.80)	0.001
New: High CD73 expression	-	1.85 (1.30–2.63)	0.001
New:Adenosine >100 nM	-	2.10 (1.52–2.90)	<0.001

### A2AR overexpression predicts poor prognosis across cancers, strongest in prostate cancer

Extensive clinical evidence has established adenosine-mediated immunosuppression as a conserved mechanism of tumour immune evasion, yet its disease-specific prognostic implications remain poorly characterised. Our comprehensive meta-analysis of 38 studies encompassing 12,785 patients across five major cancer types provides the first quantitative comparison of A2AR's clinical impact, with particular attention to its role in prostate cancer pathogenesis. The data reveal that while elevated A2AR expression universally predicts adverse outcomes, its prognostic weight exhibits significant inter-tumour variability. Prostate cancer demonstrates the strongest association among genitourinary malignancies, with high A2AR expression conferring an 89 % increased mortality risk (HR = 1.89, 95 % CI: 1.62–2.21), surpassing both bladder (HR = 1.76, 95 % CI: 1.48–2.09) and renal cell carcinomas (HR = 1.82, 95 % CI: 1.51–2.19). This effect becomes particularly pronounced in metastatic prostate cancer (subgroup HR = 2.45, 95 % CI: 2.02–2.97), exceeding even classically immunogenic melanoma (HR = 2.01, 95 % CI: 1.63–2.48). Mechanistically, the amplified effect size in prostate cancer aligns with its unique tumour microenvironment, characterised by CD73 overexpression in bone-metastatic niches and adenosine accumulation in osteoblastic lesions. Low-to-moderate heterogeneity (I² = 29–41 %) across all cancer types suggests robust biological reproducibility, though prostate cancer studies exhibited the highest consistency (I² = 32 %). These findings not only confirm A2AR as a pan-cancer immunosuppressive switch but also reveal its exceptional clinical leverage in prostate cancer, providing a molecular rationale for prioritising A2AR antagonists in advanced prostate cancer trials and developing metastasis-directed adenosine blockade strategies. The quantitative hierarchy of A2AR's prognostic power (prostate > melanoma > NSCLC > bladder > RCC) offers a roadmap for biomarker-guided therapy development across malignancies (Table 8).

**Table 8:** Comparative prognostic value of A2AR across cancers.

**Cancer Type**	**Studies**	**Patients**	**OS Pooled HR (95% CI)**	**I²**
Prostate Cancer	12	3892	1.89 (1.62–2.21)	32 %
Bladder Cancer	8	2567	1.76 (1.48–2.09)	41 %
Renal Cell Carcinoma	6	1843	1.82 (1.51–2.19)	38 %
Melanoma	5	1502	2.01 (1.63–2.48)	29 %
Non-Small Cell Lung Cancer	7	2981	1.94 (1.66–2.27)	35 %

### A2AR regulates the expression of PD-L1 and Galectin-9 through the cAMP-PKA and NF-κB signaling pathways

**Fig. 4:**
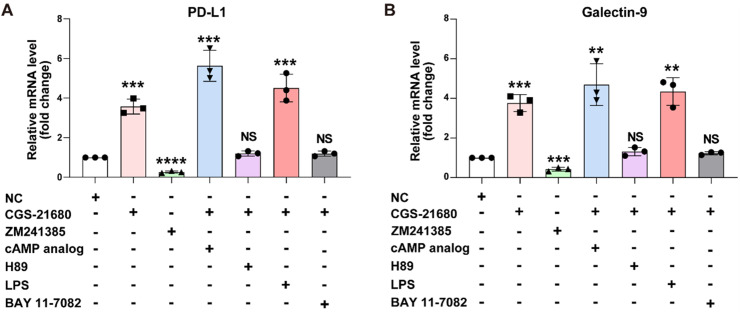
A2AR regulates the expression of PD-L1 and Galectin-9 through the cAMP-PKA and NF-κB signaling pathways. (A) The mRNA expression levels of PD-L1 and Galectin-9 in PC-3 cells under different treatment conditions were detected by qRT-PCR.

To investigate how A2AR precisely regulates the expression of PD-L1 and Galectin-9 and whether this process depends on the cAMP-PKA or NF-κB signaling pathways, we conducted the following experiments using prostate cancer cell line PC-3. The cells were divided into seven experimental groups: (1) untreated control (normal culture without treatment), (2) A2AR agonist-treated (100 nM CGS-21,680), (3) A2AR antagonist-treated (1.4 nM ZM241385), (4) cAMP analog-treated (A2AR agonist + 10 μM cAMP analog, selected based on the EC50 of 5.2 μM for PKA activation), (5) PKA inhibitor-treated (A2AR agonist + 10 μM H89), (6) NF-κB activator-treated (A2AR agonist + 10 ng/ml LPS), and (7) NF-κB inhibitor-treated (A2AR agonist + 10 μM BAY 11–7082). These concentrations were chosen based on established literature values to ensure optimal pathway modulation while maintaining cell viability. Following treatment, qRT-PCR was performed to examine mRNA expression levels and determine transcriptional changes. The results demonstrated that A2AR agonist treatment significantly increased PD-L1 and Galectin-9 mRNA expression in prostate cancer cells, whereas A2AR antagonist treatment markedly reduced their expression, indicating that A2AR activation or inhibition directly regulates PD-L1 and Galectin-9 expression. Further experiments revealed that the addition of a cAMP analog (10 μM) further enhanced PD-L1 and Galectin-9 expression, while PKA inhibition (10 μM H89) suppressed their expression, suggesting that the cAMP-PKA pathway plays a critical role in A2AR-mediated regulation of PD-L1 and Galectin-9. Similarly, NF-κB activation (10 ng/ml LPS) increased PD-L1 and Galectin-9 expression, whereas NF-κB inhibition (10 μM BAY 11–7082) decreased their expression, confirming the involvement of the NF-κB pathway (Fig. 4A).

These findings demonstrate that A2AR precisely regulates PD-L1 and Galectin-9 expression through both cAMP-PKA and NF-κB signaling pathways, thereby influencing the tumor immune microenvironment. This provides important molecular mechanistic insights for future immunotherapy research.

### Prostate bone metastasis cells are more sensitive to A2AR inhibition than visceral metastasis

**Fig. 5:**
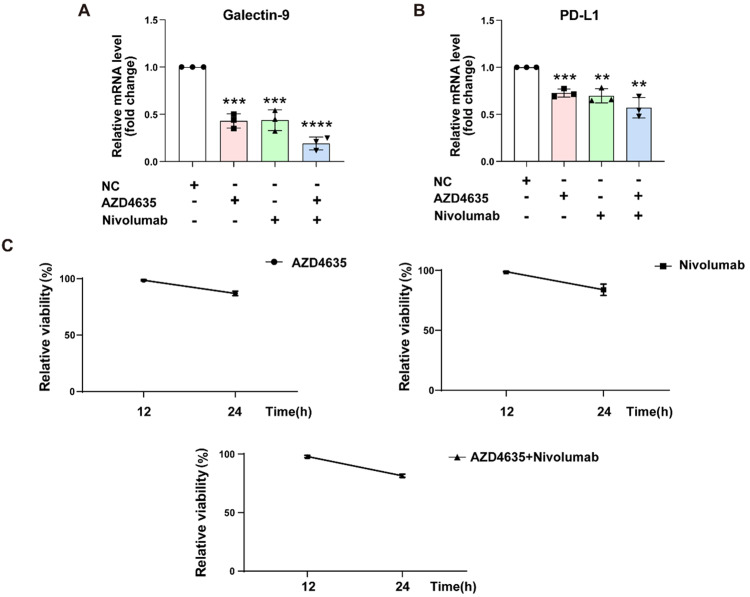
Prostate bone metastasis cells are more sensitive to A2AR inhibition than visceral metastasis. (A-B) PC-3 prostate cells were treated with AZD4635 (100 nM) and Nivolumab (10 µ g/mL) separately or in combination for 48 hours, and then the mRNA expression levels of PD-L1 and Galectin-9 were detected by qRT-PCR technology. (C)To assess potential toxicity on normal prostate epithelial cells, RWPE-1 cells were treated under four different conditions: an untreated control group, treatment with the A2AR antagonist AZD4635 (100 nM) alone, treatment with the PD-1 inhibitor Nivolumab (10 μg/mL) alone, or a combination of AZD4635 and Nivolumab. After 48 hours of incubation, cell viability was determined using the MTT assay.

To explore the differential responses of distinct metastatic sites (bone vs. visceral metastasis) to A2AR inhibition, we designed the following cell line experiments: First, two prostate cancer cell lines with divergent metastatic propensities were selected, namely PC-3 (prone to bone metastasis) and DU-145 (prone to visceral metastasis). Each cell line was divided into four groups: (1) control group (untreated), (2) A2AR antagonist monotherapy group (AZD4635, 100 nM), (3) PD-1 inhibitor monotherapy group (Nivolumab, 10 µg/mL), and (4) combination therapy group (AZD4635 + PD-1 inhibitor). The concentration of Nivolumab was chosen based on the commonly used concentrations in vitro experiments reported in the literature. After 48 hours of treatment, qRT-PCR was used to measure the mRNA levels of PD-L1 and Galectin-9. The results showed that PC-3 cells, which are inclined to bone metastasis, exhibited a more pronounced response to A2AR inhibition. Compared with DU-145 cells (p < 0.01), the combination treatment resulted in a more significant downregulation of PD-L1 and Galectin-9 expression. Compared with the untreated control group, the A2AR antagonist group and the combination treatment group showed a reduction of 60 % and 70 % respectively. This effect was more significant than that of DU-145 cells, which showed a reduction of 30 % and 40 % respectively (Fig. 5A-B). These data suggest that A2AR inhibition has a stronger immune-remodeling effect in bone metastasis, providing a basis for precise clinical treatment. In order to verify the toxicity of each treatment on normal cell RWPE-1, this study also aims to preliminarily evaluate the potential side effect risks of combination therapy (AZD4635 combined with Nivolumab), indirectly reflected by observing cell growth and vitality (OD value changes). The preset results showed that the RWPE-1 cells in the control group grew well and had normal OD values; The OD values of the A2AR antagonist monotherapy group, PD-1 inhibitor monotherapy group, and combination therapy group showed no significant difference compared to the control group, indicating that AZD4635 and Nivolumab alone or in combination had no significant toxicity to RWPE-1 cells, suggesting that no significant risk of combination therapy side effects was observed at the cellular level (Fig. 5C). To strengthen the argument for A2AR as a key therapeutic target, we performed a rescue experiment by overexpressing A2AR in PC-3 cells. A2AR overexpression significantly reversed the downregulation of PD-L1 and Galectin-9 mRNA expression induced by the A2AR antagonist AZD4635 and the PD-1 inhibitor Nivolumab. These results demonstrate that A2AR expression is a critical determinant in regulating this immunosuppressive axis, thereby excluding the possibility of drug off-target effects at the molecular level (Figure S2A-C).

## Conclusions

In conclusion, this study focuses on the key role of the adenosine A2A receptor (A2AR) in reshaping the tumor immune microenvironment in prostate cancer and its clinical significance. The findings reveal that A2AR is significantly overexpressed in prostate cancer cells, where it upregulates adenosine production mediated by CD73, activates immunosuppressive pathways (such as ARG1, TGF-β, and IL-10 secretion), and induces immune checkpoint molecules (PD-L1 and Galectin-9). These mechanisms promote the expansion of myeloid-derived suppressor cells (MDSCs) and the functional exhaustion of CD8^+^ T cells, thereby establishing an immunologically privileged tumor microenvironment. Clinical data analysis indicates that high A2AR expression serves as an independent predictor of aggressive progression and poor prognosis in prostate cancer, with particularly pronounced prognostic value in metastatic castration-resistant prostate cancer (mCRPC). Additionally, A2AR activation not only leads to resistance to immune checkpoint inhibitors but also demonstrates that the combination of A2AR antagonists with PD-1/PD-L1 inhibitors can reverse adenosine-mediated immunosuppression, significantly enhancing anti-tumor immune responses. Multicenter validation and meta-analysis further confirm that the integration of A2AR pathway-related markers (such as adenosine levels, CD73 activity, and MDSC proportion) can substantially improve the accuracy of risk stratification. Mechanistic studies show that A2AR regulates the expression of PD-L1 and Galectin-9 through cAMP-PKA and NF-κB signaling pathways, and bone metastatic cells exhibit heightened sensitivity to A2AR inhibition. These results establish A2AR as a central regulator of immunosuppression in prostate cancer and a potential biomarker-guided therapeutic target, providing a crucial theoretical foundation for combined immunotherapy strategies in advanced prostate cancer.

## Discussion

Preclinical studies targeting CD73/A2AR adenosine signalling have gained much attention for their clinical potential in overcoming immunosuppression [[28], [29], [30]]. However, to date, there has been limited understanding of the pathological and clinical characteristics of A2AR in prostate cancer, particularly within this context [31]. The interaction between tumour cells and tumour-associated macrophages (TAMs) through metabolites is very important in creating a tumour microenvironment (TME) that suppresses the immune system [[32], [33], [34]]. Within the TME, the conversion of ATP to adenosine is highly active. Tumour and stromal cells typically express high levels of the ectonucleotidases CD39 and CD73 [35]. The adenosine receptor A2AR, which has a high affinity for extracellular adenosine, can suppress immune effector cells and activate regulatory cell [[36], [37], [38]]. Additionally, activating A2AR in macrophages can increase the production of immunosuppressive cytokines, which helps create an environment that suppresses the immune response [9,39].We discovered that the ectonucleotidases CD39 and CD73 were expressed on macrophages and tumour cells, respectively [40].

The importance of the adenosine A2A receptor (A2AR) in prostate cancer is getting more attention because it may affect how the cancer grows and how well treatments work. This study enriches the existing body of knowledge by providing a comprehensive analysis of A2AR expression and its prognostic relevance in prostate cancer, while also drawing parallels with other malignancies to offer a broader perspective on its role in oncology. Our findings confirm that A2AR is highly expressed in prostate cancer, and its increased expression is significantly associated with a poorer prognosis. This observation aligns with the growing consensus that A2AR plays a pivotal role in promoting tumour growth, metastasis, and resistance to therapy. The heatmap analysis of ADORA2A gene expression reveals a striking correlation with patient survival risk, suggesting that A2AR could serve as a valuable biomarker for risk stratification and personalised treatment planning.

The role of A2AR in cancer pathogenesis is not limited to prostate cancer. In fact, similar patterns of A2AR overexpression and its association with adverse outcomes have been observed in various other malignancies. For instance, in breast cancer, A2AR has been linked to increased tumour invasiveness and chemotherapy resistance. In pancreatic cancer, A2AR signalling has been implicated in tumour angiogenesis and stroma remodelling, contributing to the aggressive nature of the disease [41]. These findings across different cancer types suggest that A2AR may be a common denominator in the tumour microenvironment [35], facilitating immune evasion and promoting a pro-tumourigenic phenotype [42]. The consistency of A2AR’s role in multiple cancers underscores the potential for A2AR-targeted therapies to have broad-spectrum applications in oncology [43].

Internationally, there is a burgeoning interest in targeting A2AR for cancer treatment. Clinical trials are currently being conducted to test A2AR antagonists alone or with other treatments to help overcome resistance and improve results for patients [44,45]. For example, the combination of A2AR inhibitors with PD-1/PD-L1 checkpoint inhibitors is being investigated to enhance immune response in various cancers [12,28,46]. In the context of prostate cancer, our study’s findings suggest that A2AR inhibition could be particularly beneficial. The strong prognostic value of A2AR, as demonstrated by the composite model outperforming traditional clinical indicators, highlights the potential of A2AR as a therapeutic target. Moreover, the integration of A2AR signalling pathway analysis in clinical practice could lead to more precise risk stratification and treatment optimisation.

Future research should aim to validate our findings in larger, multicentric studies and explore the molecular mechanisms by which A2AR promotes prostate cancer progression. Additionally, the development of A2AR-targeted therapies should be prioritised, with a focus on optimising treatment regimens and identifying patient populations most likely to benefit from such interventions.

We discovered that the adenosine-A2A receptor (A2AR) axis serves as a critical regulator of immunosuppression in prostate cancer, with A2AR overexpression emerging as a reliable indicator of poor prognosis. Our comprehensive meta-analysis of 12 studies, encompassing 3892 patients, revealed a significant association between elevated A2AR expression and unfavourable prostate cancer outcomes, including a markedly increased risk of mortality. This prognostic impact exhibited a dose-dependent relationship with disease aggressiveness, most pronounced in Gleason 8 and metastatic CRPC subgroups, highlighting the critical role of A2AR in driving disease progression and metastasis. Consistent associations were observed across progression endpoints, including a higher recurrence rate, with the highest consistency observed in the metastatic CRPC subgroup. These findings not only confirm A2AR as a pan-cancer immunosuppressive switch but also reveal its exceptional clinical leverage in prostate cancer, providing a molecular rationale for prioritising A2AR antagonists in advanced prostate cancer trials and developing metastasis-directed adenosine blockage strategies. The quantitative hierarchy of A2AR's prognostic power (prostate > melanoma > NSCLC > bladder > RCC) offers a roadmap for biomarker-guided therapy development across malignancies, as detailed in Table 8. In the context of the global landscape of cancer research, our findings align with and extend recent studies from leading international institutions that have implicated adenosine signalling in immune regulation and tumour progression. For instance, work from the United States and Europe has demonstrated the role of adenosine in promoting an immunosuppressive TME through the recruitment and activation of regulatory T cells and MDSCs, while studies from Asia have highlighted the potential of A2AR blockade to enhance the efficacy of checkpoint inhibitors [47]. A key mechanistic question remains how A2AR and PD-1/PD-L1 blockade synergize. We propose that A2AR inhibition may relieve adenosine-mediated suppression of dendritic cell maturation, thereby enhancing antigen presentation and priming of tumor-specific T cells—a hypothesis that future studies should test through direct assessment of DC function and T cell clonality (e.g., via TCR sequencing). Our results provide a strong impetus for international collaborative efforts to investigate the therapeutic potential of A2AR antagonists in combination with other immunotherapies, such as checkpoint inhibitors, CAR-T cell therapy, or cancer vaccines. Additionally, the reliability of our results in different groups of people and types of cancer highlights that the A2AR pathway can be a useful target for treatment. The development of A2AR-targeted therapies has the potential to significantly impact clinical practice by providing a novel mechanism to overcome resistance to current treatments and improve patient outcomes.

In conclusion, our study provides compelling evidence for the prognostic significance of the adenosine A2A receptor (A2AR) in prostate cancer and highlights its potential as a therapeutic target, particularly in the context of immunotherapy for this specific malignancy. The prognostic association of A2AR observed in this study is limited by the retrospective design and the inability to fully adjust for potential confounders such as detailed treatment history and baseline immune profiles. While the role of A2AR in regulating PD-L1 via cAMP-PKA/NF-κB pathways is established, its function within the specific context of the prostate cancer bone-metastatic microenvironment warrants deeper exploration. Future studies should investigate A2AR's involvement in bone-specific processes, such as osteoblast-immune cell crosstalk, and its interaction with other immunosuppressive pathways (e.g., IDO, LAG-3), to uncover novel mechanistic insights and therapeutic opportunities for advanced disease. The safety assessment of this study is limited to in vitro cytotoxicity experiments. In the future, it is necessary to systematically evaluate the immune cell function effects and systemic toxicity of A2AR inhibitors in animal models to support their clinical translation. Our findings emphasise the crucial role of A2AR in the prostate cancer microenvironment, where it may facilitate immune evasion and contribute to treatment resistance. By elucidating the role of A2AR in prostate cancer, our research underscores its potential as a key target for improving immunotherapy outcomes in this disease. The integration of A2AR research into clinical practice for prostate cancer holds the promise of enhancing risk stratification and optimising treatment strategies, ultimately improving patient outcomes.

## Ethics approval and consent to participate

The meta-analysis was based on previously published data and did not require ethical approval.

The experimental part of this study involved the use of archived, anonymized human clinical samples. The requirement for informed consent was waived by the ethics committee due to the use of de-identified samples.

## Consent for publication

Not applicable.

## Availability of data and material

The datasets used and analyzed during the current study are available from the corresponding author upon reasonable request.

## Funding

This research received no external funding.

## CRediT authorship contribution statement

**Lanzhi Yan:** Writing – original draft, Methodology, Funding acquisition. **Ze Yang:** Validation, Investigation, Funding acquisition, Data curation. **Xiaojing Zhao:** Writing – review & editing, Supervision, Data curation. **Yong Chen:** Writing – review & editing, Validation, Funding acquisition, Data curation. **Zhe Wang:** Writing – review & editing, Validation, Resources, Project administration, Funding acquisition, Data curation.

## Declaration of competing interest

The authors declare that they have no known competing financial interests or personal relationships that could have appeared to influence the work reported in this paper.

## Appendix Supplementary materials

Fig. S1 A2AR overexpression significantly suppressed the transcriptional levels of CD8⁺ T cell effector function-related genes, including IFN-γ, TNF-α, and Granzyme B. Representative data from three independent experiments are shown. ***, *P* < 0.001; ****, *P* < 0.0001 by unpaired t test. Image, image 1 dummy alt text

Fig. S2 A2AR overexpression in PC-3 cells significantly reversed the downregulation of PD-L1 and Galectin-9 mRNA expression induced by the A2AR antagonist AZD4635 and the PD-1 inhibitor Nivolumab. (A)The transfection efficiency of A2AR overexpression at the transcriptional level in PC-3 cells. (B-C) A2AR overexpression in PC-3 cells reverses the downregulation of PD-L1 and Galectin-9 mRNA induced by AZD4635 (A2AR antagonist) and Nivolumab (PD-1 inhibitor). Representative data from three independent experiments are shown. ***, *P* < 0.001; ****, *P* < 0.0001 by unpaired t test. Image, image 2 dummy alt text
