# Supplementary figures and images for: A2AR as a key target for immune microenvironment remodeling in prostate cancer
Source: Transl Oncol. 2026 Feb 27;66:102720. doi: 10.1016/j.tranon.2026.102720 (PMC12963924; doi:10.1016/j.tranon.2026.102720)

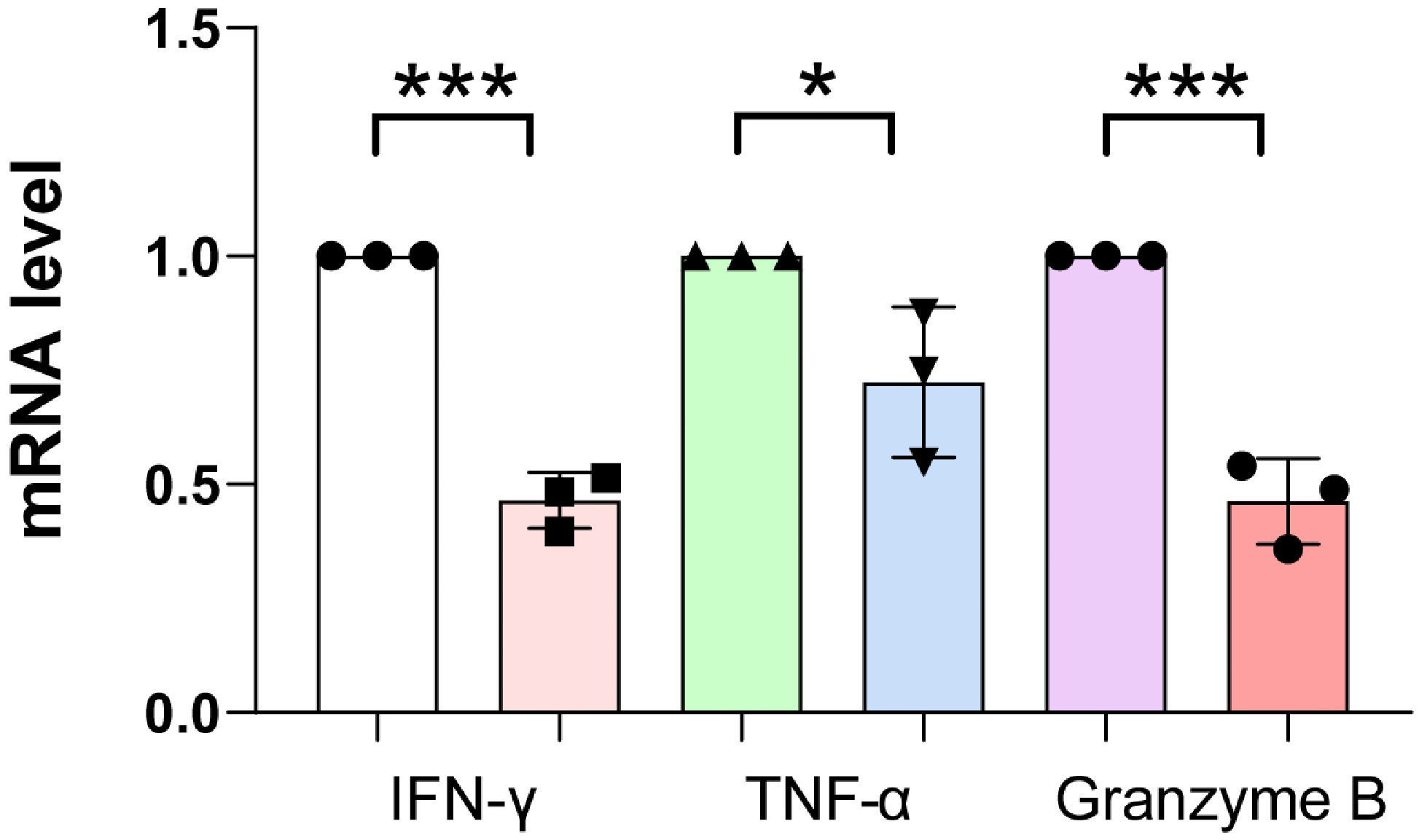

Supplement: Supplementary file 1 — Fig. S1 A2AR overexpression significantly suppressed the transcriptional levels of CD8⁺ T cell effector function-related genes, including IFN-γ, TNF-α, and Granzyme B. Representative data from three independent experiments are shown. ***, P < 0.001; ****, P < 0.0001 by unpaired t test. [file mmc1.jpg]

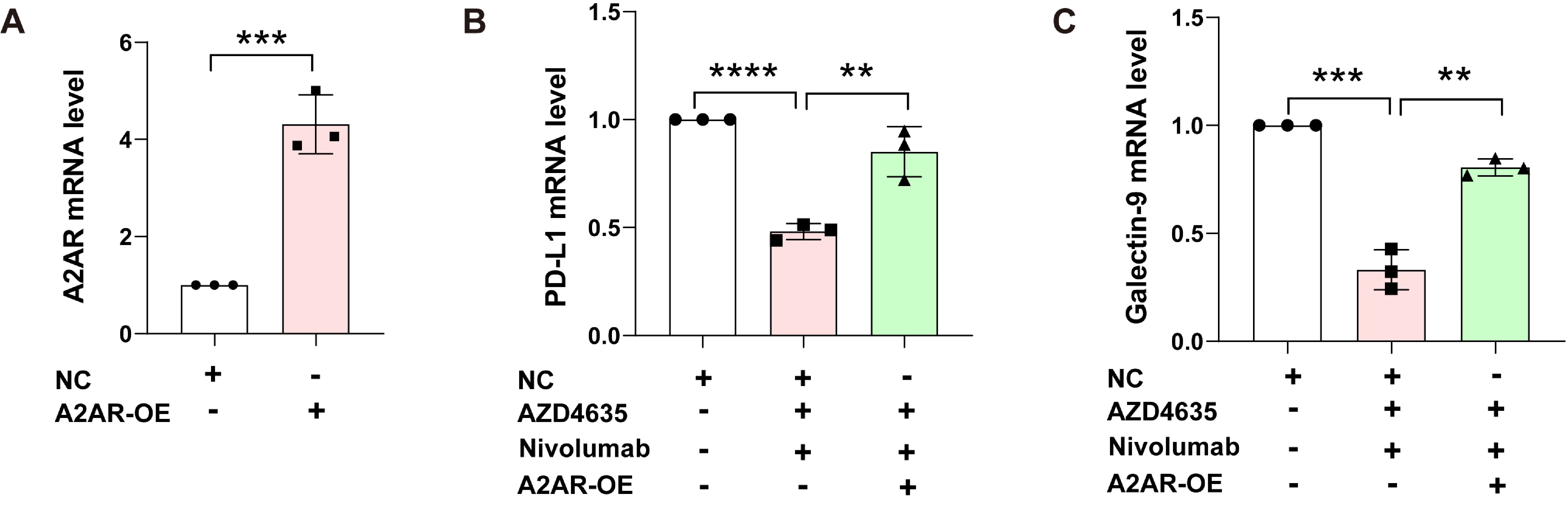

Supplement: Supplementary file 2 — Fig. S2 A2AR overexpression in PC-3 cells significantly reversed the downregulation of PD-L1 and Galectin-9 mRNA expression induced by the A2AR antagonist AZD4635 and the PD-1 inhibitor Nivolumab. (A)The transfection efficiency of A2AR overexpression at the transcriptional level in PC-3 cells. (B-C) A2AR overexpression in PC-3 cells reverses the downregulation of PD-L1 and Galectin-9 mRNA induced by AZD4635 (A2AR antagonist) and Nivolumab (PD-1 inhibitor). Representative data from three independent experiments are shown. ***, P < 0.001; ****, P < 0.0001 by unpaired t test. [file mmc2.jpg]
